# Glycyrrhizin arginine salt protects against cisplation-induced acute liver injury by repressing BECN1-mediated ferroptosis

**DOI:** 10.3389/fphar.2023.1219486

**Published:** 2023-09-06

**Authors:** Jun Guo, Jiameng Yin, Pu Liu, Xin Zhang, Jie Wei, Mingjun Wang, Yanxia Xiao, Yongzhan Zhen, Yajun Lin, Jian Li

**Affiliations:** ^1^ The Key Laboratory of Geriatrics, Beijing Hospital/National Center of Gerontology of National Health Commission, Beijing Institute of Geriatrics, Institute of Geriatric Medicine, Chinese Academy of Medical Sciences, Beijing, China; ^2^ Hebei Key Laboratory for Chronic Diseases, School of Basic Medical Sciences, North China University of Science and Technology, Tangshan, China; ^3^ College of Basic Medical Sciences, Capital Medical University, Beijing, China

**Keywords:** glycyrrhizin arginine salt, cisplatin, liver injury, BECN1-xCT complex, ferroptosis

## Abstract

The study aimed to investigate the protective effects and biological mechanisms of glycyrrhizin arginine salt (Gly-Arg) against cisplatin (Cis)-induced liver injury. Our data showed that Gly-Arg improved Cis-induced liver injury. Further study showed that BECN1 (beclin1) and LC3-II/LC3-I protein expression was significantly increased in primary hepatocytes and mouse liver tissues after Cis treatment, but Gly-Arg reduced the protein levels of BECN1 and LC3-II/LC3-I in primary hepatocytes and mouse liver tissues. Also, Gly-Arg improved indicators related to Cis-induced ferroptosis. Furthermore, Cis increased colocalization of lysosomal membrane-associated protein 1A (LAMP1) with ferritin heavy chain 1 (FTH1) in primary mouse hepatocytes, while Gly-Arg intervention attenuated this colocalization in primary hepatocytes. More improtantly, Cis enhanced the formation of the BECN1-xCT complex, thus inhibiting solute carrier family 7 member 11 (SLC7A11, xCT) and glutathione peroxidase-4 (GPX4) activity. In contrast, Gly-Arg intervention disrupted the formation of this complex. However, Gly-Arg alleviated Cis-induced liver injury in mice by preventing autophagic death and ferroptosis through the inhibition of BECN1-xCT complex formation.

## Introduction

Cisplatin (Cis) is a widely used chemotherapeutic agent in oncology, but its toxic effects on several organs, especially the liver and kidney, have largely limited its clinical application (Gong et al., 2021; Kurt et al., 2021). The exact mechanism of Cis-induced hepatotoxicity is not yet fully understood. Some data show that Cis can cause ultrastructural changes in liver tissue and participate in the death of hepatocytes (Niu et al., 2017; El Nashar et al., 2021; Kurt et al., 2021). Once liver cells are damaged, various biological functions are severely dysregulated, and human health is vulnerable (El Nashar et al., 2021). Therefore, understanding the mechanism of liver injury caused by Cis and preventing its toxic side effects are urgent issues to be addressed.

Programmed cell death (PCD) is a process that is essential for the maintenance of the physiological activity of cells (D'Arcy, 2019). Apoptosis is the most common form of PCD (D'Arcy, 2019). Recently, several forms of PCD have been discovered, such as autophagy-dependent death, necroptosis, and ferroptosis (D'Arcy, 2019; Griffioen and Nowak-Sliwinska, 2022; Mou et al., 2019). Among them, ferroptosis is characterized by iron-dependent and lipid reactive oxygen species (ROS) accumulation that does not depend upon caspase-mediated cell death (Mou et al., 2019). Morphologically, a typical feature of ferroptosis includes mitochondrial volume reduction, mitochondrial cristae decrease and disappearance, outer membrane fragmentation, and decreased mitochondrial membrane potential (MMP) (Gao et al., 2019). Glutathione peroxidase-4 (GPX4) is a widespread peroxidation inhibiting enzyme that catalyzes the conversion of harmful lipid peroxides into nontoxic alcohols (Xie et al., 2016). Key features of ferroptosis include inhibition of GPX4 enzyme activity, reduced glutathione (GSH) synthesis, and accumulation of lipid peroxides (Xie et al., 2016). Additionally, metabolic homeostasis of intracellular iron is important for the regulation of ferroptosis (Jiang et al., 2021). Cellular iron homeostasis (iron import, storage, utilization and export) is primarily maintained by genes related to iron metabolism (Chen et al., 2021). For example, downregulation of ferritin heavy chain 1 (FTH1) expression increases the cellular Fe^2+^ content, raises the level of intracellular oxidative stress and thus induces ferroptosis (Fang et al., 2021).

Increasing evidence has shown that autophagy is required for ferroptosis and that certain regulators of autophagy (e.g., BECN1, also known as Beclin1) play an important role in the process of ferroptosis (Hou et al., 2016; Liu et al., 2020). BECN1 is a key regulator of autophagy and is involved in the regulation of mammalian autophagosome formation (Guo et al., 2019). It is phosphorylated by protein kinase AMP-activated catalytic subunit alpha 1 (AMPK) at the Ser90/93/96 site and interacts with the system Xc-core protein component of solute carrier family 7 member 11 (SLC7A11, xCT)) to form a BECN1-xCT complex that directly inhibits system Xc-activity and promotes ferroptosis in cells (Song et al., 2018; Guo et al., 2019; Lee et al., 2022). Moreover, knockdown of the BECN1 gene attenuates the inhibitory effect of ferroptosis inducers (e.g., erastin and sorafenib) on system Xc-activity (Song et al., 2018; Zhang et al., 2020). Overall, these studies suggest that BECN1 regulates ferroptosis by modulating autophagic activity.

Glycyrrhizin (Gly), a triterpenoid extracted from licorice roots, is a safe and nontoxic natural product with a wide range of pharmacological effects in the treatment of cancer, inflammatory conditions, and viral diseases (Li et al., 2018). However, Gly forms a highly viscous gel solution when exposed to water, which affects its use *in vivo*. Glycyrrhizin arginine salt (Gly-Arg) is formed by mixing Gly with arginine (1:2 M ratio) in water, and this complex can effectively block the formation of the macromolecular polymerization gel of Gly (Zhang et al., 2018). It was found that Gly-Arg improves cholestatic liver injury by suppressing liver fibrosis (Zhang, H. et al., 2018). However, the amelioration of cisplatin-induced acute liver injury by Gly-Arg and the underlying mechanism have not been reported. In this study, we established mouse and liver hepatocyte models of Cis-induced acute liver injury and intervened with Gly-Arg, so as to investigate whether Gly-Arg could ameliorate Cis-induced acute liver injury and the specific molecular mechanisms.

## Materials and methods

### Reagents

3-(4,5-Dimethylthiazol-2-yl)-2,5-diphenyltetrazolium bromide (MTT) and dimethyl sulfoxide (DMSO) were purchased from Sigma Aldrich (USA). Gly (98% purity) was purchased from Nanjing Corsas Medical Technology (Nanjing, China), Cis was purchased from Qilu Pharmaceutical (Jinan, China) Co., and arginine was purchased from Lablead Biotech, Co., Ltd. (Beijing, China). An aspartate aminotransferase (AST/GOT) test kit (C010-2-1), alanine aminotransferase (ALT/GPT) assay kit (C009-2-1), malondialdehyde (MDA) assay kit, catalase (CAT) assay kit, glutathione peroxidase (GSH-PX) assay kit and lactate dehydrogenase (LDH) assay kits were purchased from Nanjing Jiancheng Institute of Biological Engineering (Nanjing, China). Intracellular iron colorimetric assay kits were purchased from Wuhan Elabscience Biotechnology Co. Acridine orange (AO) assay kit, mitochondrial membrane potential (JC-1) assay kit, C11 BODIPY 581/591 lipid peroxidation fluorescent probe, mitochondrial reactive oxygen species (Mito-ROS) probe and intracellular ferroOrange fluorescent probe were purchased from Sigma‒Aldrich, Abcam, Invitrogen and Beiren Chemical Technology (Beijing) Ltd. BECN1, *β*-actin, FTH1, GPX4 and xCT antibodies were purchased from Cell Signaling Technology (Danvers, MA, US). LC3 antibody was purchased from Sigma‒Aldrich (L8918). LAMP1 antibody was purchased from SANTA CRUZ BIOTECHCHNOLOGY, INC. A glutamate release kit was purchased from Thermo Fisher Scientific (A12221, MA, US).

### Animals

Forty specific pathogen-free (SPF) 8-week-old male C57BL/6J mice (weighing 20–25 g) were purchased from Beijing Bochiyuan Biotechnology Co., Ltd. (Beijing, China). All animals were housed in a light-controlled room (12 h light/dark cycle) with an ambient temperature of 25°C and free access to drinking water and standard food. All animals were humanely cared for according to institutional guidelines. This experiment was approved by the Animal Ethics Committee of North China University Of Science And Technology (2021-SY-043).

The animals (*n* = 40) were randomly divided into four groups (*n* = 10 for each group): the control group, Cis group, Cis + Gly-Arg low-dose treatment group (L-Gly-Arg, 100 mg/kg once every day for 7 days) and Cis + Gly-Arg high-dose treatment group (H-Gly-Arg, 200 mg/kg once every day for 7 days). The pharmacological Cis-induced liver injury model was constructed by intraperitoneal injection of Cis 20 mg/kg once in the Cis group 72 h before execution. The control group was injected with saline only. For the L-Gly-Arg and H-Gly-Arg groups, 100 mg/kg and 200 mg/kg Gly-Arg were given once every day for 7 days before the induction of acute liver injury models. Blood and liver tissue samples were collected for the subsequent studies.

### Isolation and culture of primary mouse hepatocytes

Hepatocytes were isolated from mice by two-step collagenase perfusion as previously described (Storey et al., 2012; Fukuoka et al., 2017; Matsumura et al., 2019). First, after perfusion of Hanks’ balanced salt solution (HBSS, Sigma‒Aldrich) containing ethylene glycol tetraacetic acid without Ca or Mg was perfused at a rate of 3–4 mL/min through the portal vein. When the color of the liver was observed to turn beige or light brown, the liver was perfused with .3 mg/mL collagenase (type IV; Sigma Aldrich) in HBSS by the same route and at the same rate until the liver structure was significantly degraded. The isolated cells were suspended in high-glucose DMEM (Cyclone Utah, United States) containing 10% fetal bovine serum (FBS). Cells were then filtered through 100 sieve wells and purified by centrifugation (500 x g, 5 min) at 4°C. Hepatocyte viability was assessed by a trypan blue exclusion assay. Cells were incubated overnight at 37°C in a CO_2_ incubator before being used for experiments.

### Cell viability assays

Cells (3 × 10^3^ cells/well) were seeded in 96-well plates and incubated overnight at 37°C and treated with 1 µM Fe^2+^ inhibitor (Fer-1), 10 µM deferiprone (DFO), 10 µM 3-methyladenine (3-MA), 10 µM chloroquine (CQ), 10 µM necrostatin-1 (Nec-1), 10 µM Z-VAD-FMK (ZVAD) and 25, 50, 100, 150, or 200 µM Gly-Arg. Different concentrations of Cis were added for 24 h, followed by the addition of 5 mg/mL MTT solution (20 µL/well), and then the cells were incubated at 37°C in the dark for 4 h. Finally, the supernatant was removed, and 150 µL DMSO was added. Cell viability was measured at OD490 nm by a Multiskan™ microplate reader (Thermo Fisher Scientific, Inc., USA). IC50 values were calculated using GraphPad Prism 8.4 software (GraphPad software, Inc., USA).

### Hematoxylin and eosin (H&E) staining

Four micromolar thick tissue sections were dewaxed and treated with xylene. The sections were then sequentially stained with hematoxylin (3 min) and eosin (45 s). After washing with ddH_2_O, sections were dehydrated with a concentration gradient of ethanol, treated with xylene, sealed with coverslips, and observed by light microscopy (Olympus, Japan).

### Serum biochemical analysis

Serum glutamate aminotransferase (ALT), glutamate aminotransferase (AST) and lactate dehydrogenase (LDH) were measured using commercial reagent kits (Nanjing Built) according to the manufacturer’s instructions.

### Immunofluorescence (IF) staining

The differently treated hepatocytes were fixed in 4% paraformaldehyde. The cells were then incubated overnight at 4°C with the following primary antibodies: anti-LAMP1 (Santa Cruz, sc-19992, 1:100), anti-FTH1 (CST, 4393, 1:100), anti-4-HNE antibody (Bioss Antibodise, bs-6313R; 1:100), anti-xCT (CST, 1269; 1:100), and anti-BECN1 (Proteintech, 66665-1). Subsequently, the cells were washed with PBS and incubated with FITC- and TRITC-coupled secondary antibodies and then examined by fluorescence microscopy (Keyence, Osaka, Japan). For liver tissue immunofluorescence, 4 µm thick frozen sections were prepared from the different experimental groups. The sections were washed with PBS and immersed in 3% BSA to block the nonspecific background. Sections were then incubated with rabbit polyclonal anti-4-HNE, -LAMP1, -FTH1, -XCT or -BECN1 primary antibodies at 4°C overnight. After washing 3 times with PBS, secondary antibodies were incubated for 2 h at room temperature. Sections were rerinsed with PBS, sealed with coverslips and observed under a fluorescence microscope (Keyence, Osaka, Japan).

### Immunohistochemical (IHC) analysis

Four-micrometer-thick paraffin-embedded liver tissue sections were heated to 60°C for 40 min and then subjected to xylene dewaxing and gradient ethanol hydration. The sections were blocked with 3% hydrogen peroxide and subjected to antigen repair. Next, the slides were washed with PBS 3 times for 5 min each, incubated with CD68 antibody (1:200) (CST, 97778; 1:100) overnight at 4°C, and then incubated with HRP-coupled secondary antibody for 30 min at room temperature. After washing with PBS, sections were treated with DAB solution for 20–35 s to generate peroxidase reaction products. After hematoxylin restaining, gradient ethanol dehydration, xylene transparency, and sealing, the sections were observed under a light microscope (Keyence, Osaka, Japan).

### Western blot

The liver tissue was cut into 1 mm^3^ sections, and the cells were lysed with RIPA buffer. After centrifugation at 12,000 rpm for 20 min, the supernatant was collected. The protein concentration in the supernatant was determined using the Pierce BCA Protein Assay Kit (23225, Thermo Fisher). Equal amounts of proteins (20 μg/lane) were separated by 10% SDS‒PAGE and then transferred to PVDF membranes. The PVDF membranes were then incubated at 4°C with the following primary antibodies: anti-LC3 (CST, 4599; 1:1000), anti-BECN1 (CST, 3495; 1:1000), anti-FTH1 (CST, 4393; 1:1000), anti-GPX4 (CST, 52455; 1:1000), anti-xCT (CST, 12691; 1.1000), anti-cleaved (c)-caspase3 (CST, 9664, 1:1000) and anti-β-actin (CST, 4970; 1:1000). PVDF membranes were washed with TBST buffer (137 mM NaCl, 2.7 mM KCl, 16.5 mM Tris, pH 7.4, containing 0.1% Tween-20) and incubated with secondary antibodies at room temperature for 2 h. After washing again with TBST, protein bands in the blots were detected using an ECL chemiluminescence system (GE Healthcare, US). *β*-actin was used as an internal control.

### Evaluation of mitochondrial membrane potential (MMP-1) and ROS in primary mouse hepatocytes

Primary hepatocytes were analyzed for MMP using the JC-1 staining method. In brief, primary hepatocytes were incubated with a JC-1 probe at a final concentration of 20 μM for 10 min at 37°C in the dark. Mitochondrial reactive oxygen species (mito-ROS) were measured using a final concentration of 5 μM MitoSOX™ red mitochondrial superoxide indicator (MitoSOX™, 5 μM, Invitrogen. M36008) at 37°C for 10 min in the dark. Cells were washed with PBS and immediately observed by fluorescence microscopy (Keyence, Osaka, Japan).

### Assessment of lysosomal permeability (LMP) in primary mouse hepatocytes

The LMP of hepatocytes was determined by acridine orange (AO) staining. Hepatocytes were incubated with AO (4 μg/mL; Sigma‒Aldrich, A6014) for 20 min at 37°C in the dark. The stained cells were washed twice with 3% FCS/PBS and immediately observed by fluorescence microscopy (Keyence, Osaka, Japan).

### Transfection of mRFP-GFP-LC3 adenovirus vectors

Primary hepatocytes were seeded at a density of 1 × 10^5^ cells in six-well plates at 37°C in a 5% CO_2_ incubator overnight, and then cells were transfected with mRFP-GFP-LC3 adenovirus (Hanbio Biotechnology, Shanghai, China) at a multiplicity of infection (MOI) of 30 for 24 h, followed by Cis and Gly-Arg for another 24 h. The average number of GFP-LC3 and RFP-LC3 puncta per cell was counted under a fluorescence microscope (Keyence, Osaka, Japan). When red and green fluorescence were combined, yellow puncta represented autophagosomes, and red puncta represented autolysosomes (AL).

### Transmission electron microscopy (TEM)

After conventional sampling, double fixation, dehydration, immersion, embedding, sectioning (1 mm thickness), and double staining with uranyl acetate and lead citrate, the liver tissue was observed by TEM as previously described (Cai et al., 2022).

### Lipid ROS assay

Lipid ROS was measured using the C11 BODIPY 581/591 probe (RM02821, ABclonal, US). Isolated primary hepatocytes were seeded in 6-well plates and pretreated with 1 μM Fer-1, 10 μM DFO, and 100 μM Gly-Arg for 2 h as described above and then incubated with 10 μM BODIPY 581/591 C115 for 1 h in the dark after 24 h of Cis treatment. Following three washes in PBS, the slides were observed under a fluorescence microscope (Keyence, Osaka, Japan).

### FerroOrange staining

In brief, primary hepatocytes were pretreated with Fer-1, DFO, and Gly-Arg for 2 h, treated with Cis for 24 h, incubated with a final concentration of 1 μM FerroOrange (Dojindo, F374) at 37°C in a 5% CO_2_ incubator in the dark for 30 min and immediately observed by fluorescence microscopy (Keyence, Osaka, Japan).

### Glutamate release assay

Glutamate release from primary hepatocytes into the extracellular medium was detected using the Amplex Red Glutamate Release Assay Kit (Thermo Fisher Scientific, A12221) according to the manufacturer’s instructions.

### MDA, CAT and GSH-PX quantification analysis

MDA, CAT and GSH-PX were measured using the Micro Malondialdehyde (MDA) Assay Kit, Catalase (CAT) Assay Kit, and Glutathione Peroxidase (GSH-PX) Assay Kit (Nanjing Jiancheng, Nanjing, China), respectively, according to the manufacturer’s requirements.

### Statistical analysis

All data analyses were performed using GraphPad Prism version 8.4.2 software. Data are expressed as the mean ± standard deviation. Comparisons between two groups were made using the *t*-test. One-way analysis of variance (ANOVA) was used for studies with more than two groups. *p* values <0.05 were considered to indicate statistically significant differences.

## Results

### Gly-Arg protects against pharmacological Cis-induced liver injury

**FIGURE 1:**
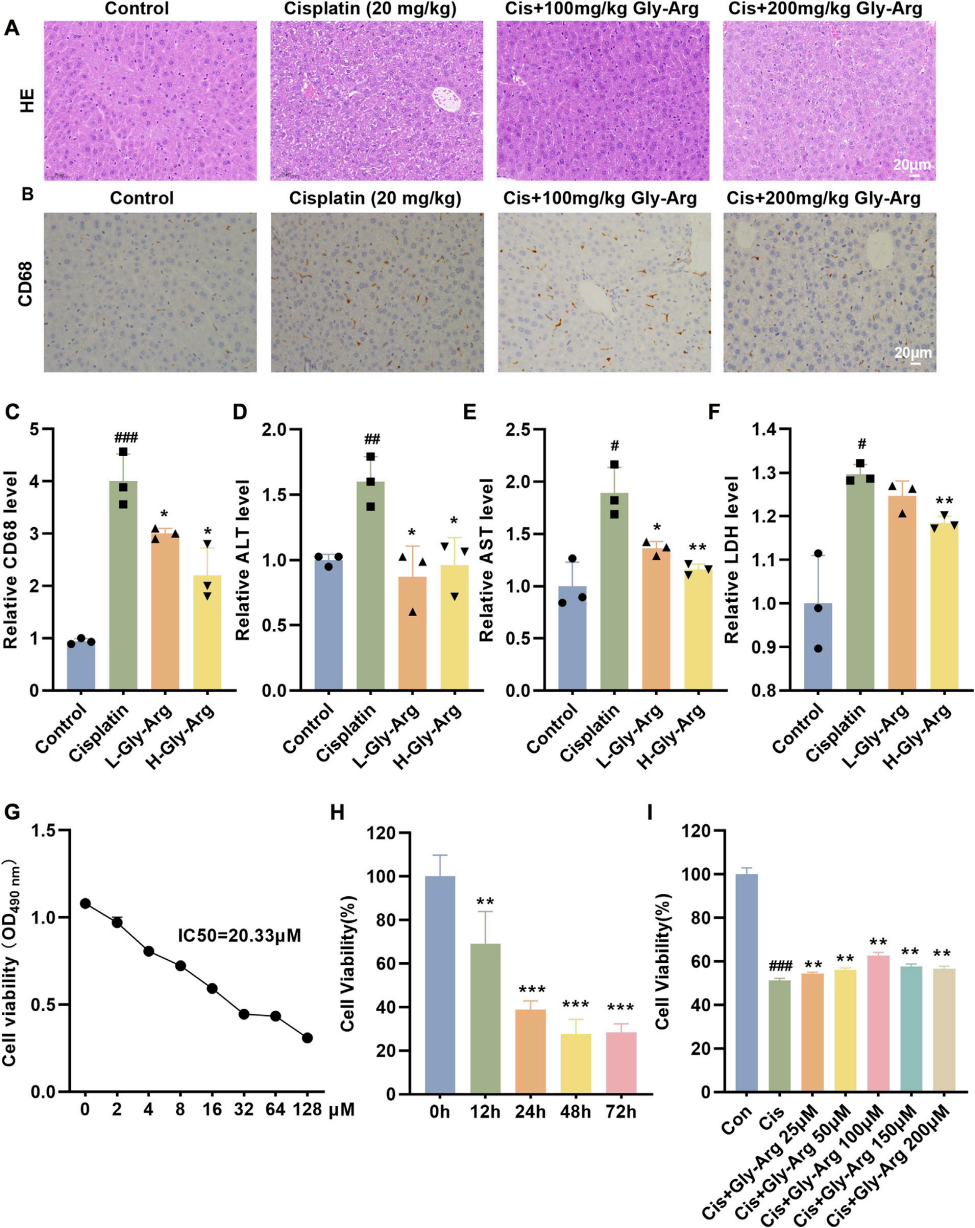
Gly-Arg protects against Cis-induced acute liver injury. **(A)** Representative H&E images of the four groups demonstrated that L-Gly-Arg and H-Gly-Arg improved Cis-induced pathological damage in the livers of mice. **(B, C)** IHC staining and statistical analysis showed that L-Gly-Arg and H-Gly-Arg decreased the expression of CD68 in the livers of mice. Compared with Cis treatment alone, Gly-Arg decreased the levels of ALT **(D)**, AST **(E)** and LDH **(F)** in the livers of mice. The MTT assay revealed that Cis treatment decreased the viability of primary hepatocytes in a concentration- **(G)** and time **(H)**-dependent manner. **(I)** The MTT assay demonstrated that Gly-Arg could reverse the decrease in cell survival caused by Cis. #*p* < 0.05, ##*p* < 0.01, ###*p* < 0.001 vs. Control; *p < 0.05, **p < 0.01, ***p < 0.001 vs. Cis.

In the livers of mice in the Cis group, pathological damage (such as variable morphology and size of hepatocytes) appeared, inflammatory cell infiltration was significantly increased, and serum ALT, AST and LDH levels were elevated. In comparison, the elevated ALT, AST and LDH levels in the Gly-Arg group were recovered, and histological changes were reversed, with the H-Gly-Arg group showing better effects than the L-Gly-Arg group (Figures 1A–F). The MTT assay revealed that Cis treatment decreased the viability of primary hepatocytes in a concentration- and time-dependent manner (Figures 1G, H). We further found that different concentrations of Gly-Arg could reverse the decrease in cell survival caused by Cis treatment, especially at 100 μM (Figure 1I). Next, we investigated the pathway Cis might induce primary hepatocyte death through. It was found that the addition of Fer-1 (a lipid peroxidation scavenger), DFO (an Fe^2+^ chelator), 3-MA (an autophagy inhibitor) and ZVAD (an apoptosis inhibitor) could reverse the Cis-induced decrease in cell viability to some extent relative to Cis treatment alone (Supplementary Figure S1). However, the addition of CQ and nec-1 (necrosis inhibitor) did not restore the Cis-induced decrease in cell viability of primary hepatocytes (Supplementary Figure S1). Therefore, we speculated that Cis treatment induces hepatocyte injury through apoptosis, iron death and autophagy pathways. Next, we investigated the specific mechanism underlying Gly-Arg amelioration of Cis-induced liver injury.

### Gly-Arg alleviates pharmacological Cis-induced liver injury by inhibiting autophagy

**FIGURE 2:**
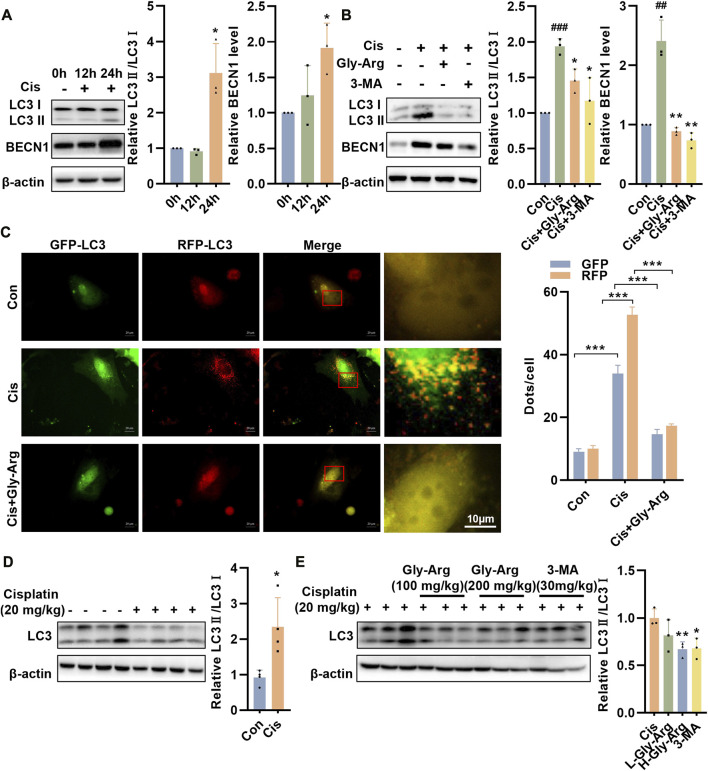
Gly-Arg alleviates pharmacological Cis-induced liver injury by inhibiting autophagy. **(A)** Western blot assay revealed that the expression levels of LC3II/LC3I and BECN1 were elevated after 24 h of Cis treatment in primary hepatocytes compared with the control group. **(B)** After pretreatment with Gly-Arg and an autophagy inhibitor (3-MA) for 2 h, LC3II/LC3I and BECN1 expression levels were decreased in primary hepatocytes compared with Cis treatment alone. **(C)** Transfection with mRFP-GFP-LC3 adenovirus demonstrated that Gly-Arg significantly reduced Cis-induced yellow puncta and red puncta. **(D)** Western blot assays showed that LC3-II/LC3-I expression was significantly higher in the Cis-induced acute liver injury mouse model than in the control group. **(E)** L-Gly-Arg and H-Gly-Arg significantly reduced the ratio of LC3-II/LC3-I in mouse liver tissues compared with the Cis-induced liver injury mouse model. ##*p* < 0.01, ###*p* < 0.001 vs. Control; *p < 0.05, **p < 0.01, ***p < 0.001 vs. Cis.

In the livers of Cis group mice, the level of an apoptosis marker, c-caspase 3, was elevated, but Gly-Arg did not significantly decrease the level of c-caspase 3, indicating that Gly-Arg did not alleviate Cis-induced hepatocyte apoptosis (Supplementary Figure S2). Next, we examined whether Gly-Arg alleviated Cis-induced liver injury by inhibiting autophagy. Western blot assays revealed that the expression levels of LC3II/LC3I and BECN1 were elevated after 24 h of Cis treatment in primary hepatocytes compared with those of the control group, suggesting that autophagy increased in primary hepatocytes after Cis treatment (Figure 2A). However, after pretreatment with Gly-Arg and an autophagy inhibitor (3-MA) for 2 h, LC3II/LC3I and BECN1 expression levels were decreased in primary hepatocytes compared with those of the Cis group (Figure 2B). The fusion of autophagic vesicles and lysosomes has an important role in autophagic flux, and sustained autophagic flux leads to autophagic death (Mizushima et al., 2010). Transfection of mRFP-GFP-LC3 adenovirus demonstrated that Cis treatment enhanced the formation of yellow puncta and red puncta, suggesting that it activated autophagic flux in primary hepatocytes (Figure 2C). In contrast, Gly-Arg significantly reduced Cis-induced yellow puncta and red puncta, indicating that Gly-Arg blocked autophagic flux in primary hepatocytes (Figure 2C). Moreover, LC3-II/LC3-I expression was significantly higher in the Cis-induced acute liver injury mouse model than that of the control group (Figure 2D). L-Gly-Arg and H-Gly-Arg significantly reduced the ratio of LC3-II/LC3-I in mouse liver tissues compared with the Cis-induced liver injury mouse model (Figure 2E). Therefore, we hypothesized that Gly-Arg could inhibit Cis-induced excessive autophagy activation.

### Gly-Arg rescues pharmacological Cis-induced liver injury by inhibiting ferroptosis

**FIGURE 3:**
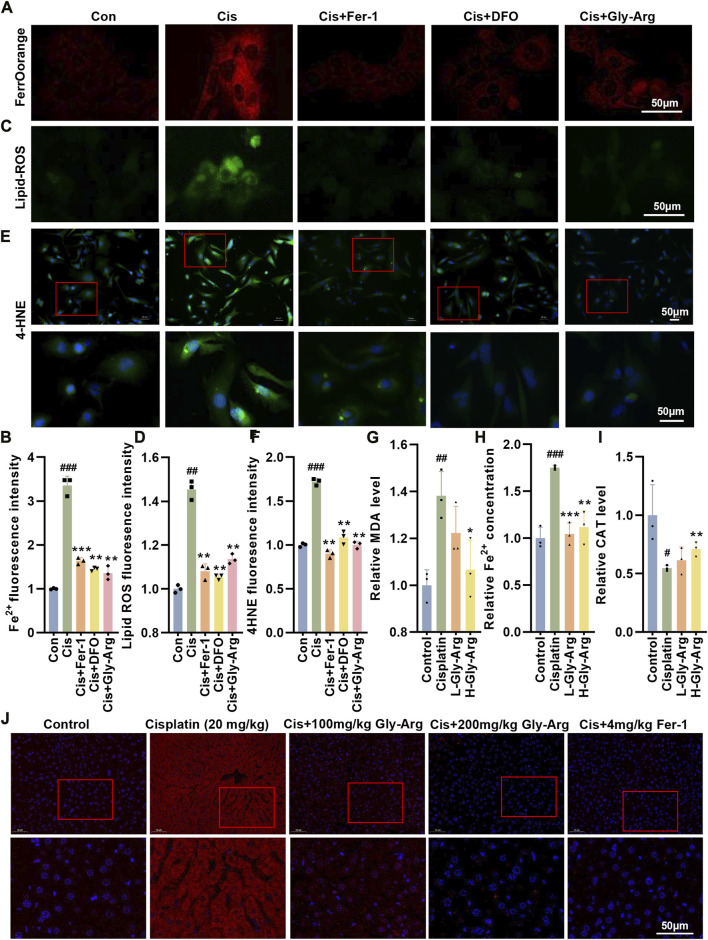
Gly-Arg rescues pharmacological Cis-induced liver injury by inhibiting ferroptosis. **(A, B)** FerroOrange staining showed that the levels of Fe^2+^ were significantly lower in hepatocytes cotreated with Fer-1, DFO and Gly-Arg compared with Cis treatment alone. **(C, D)** C11 BODIPY 581/591 probe detection demonstrated that the levels of lipid ROS in the primary hepatocytes were reduced after cotreatment with Fer-1, DFO, Gly-Arg and Cis compared with Cis treatment alone. **(E, F)** IF staining showed that the production of 4-HNE was significantly reduced after pretreatment with Fer-1, DFO, and Gly-Arg in contrast to Cis treatment alone. Gly-Arg alleviated Cis-induced upregulation of MDA **(G)** and Fe2+ **(H)** and downregulation of CAT **(I)** in the mouse livers. **(J)** IF staining showed that L-Gly-Arg and H-Gly-Arg were able to downregulate the accumulation of 4-HNE. *p < 0.05, **p < 0.01, ***p < 0.001 vs. Control; *p < 0.05, **p < 0.01, ***p < 0.001 vs. Cis.

Next, we investigated whether Gly-Arg rescued Cis-induced ferroptosis in primary hepatocytes. Compared to the control group, Fe^2+^ was significantly increased in primary hepatocytes after Cis treatment; however, the levels of Fe^2+^ were significantly lower in hepatocytes cotreated with Fer-1, DFO and Gly-Arg (Figures 3A, B). Moreover, the levels of lipid ROS in the primary hepatocytes were reduced after cotreatment with Fer-1, DFO, Gly-Arg and Cis compared with the Cis treatment alone (Figures 3C, D). In addition, the production of 4-hydroxynonenal (4-HNE), one of the major products of lipid peroxidation, was significantly reduced after pretreatment with Fer-1, DFO, and Gly-Arg in contrast to Cis treatment alone (Figures 3E, F). Additionally, the levels of MDA and Fe^2+^ were increased (Figures 3G, H), and CAT levels were decreased (Figure 3I), in the livers of mice in the Cis-group whereas advanced intervention with Gly-Arg alleviated these indices. IF staining showed that the accumulation of the lipid peroxidation product 4-HNE was increased in the Cis-group, whereas L-Gly-Arg and H-Gly-Arg were able to downregulate the accumulation of 4-HNE (Figure 3J). These results suggest that Gly-Arg rescues pharmacological Cis-induced liver injury by inhibiting ferroptosis.

### Gly-Arg reverses Cis-induced mitochondria injury

**FIGURE 4:**
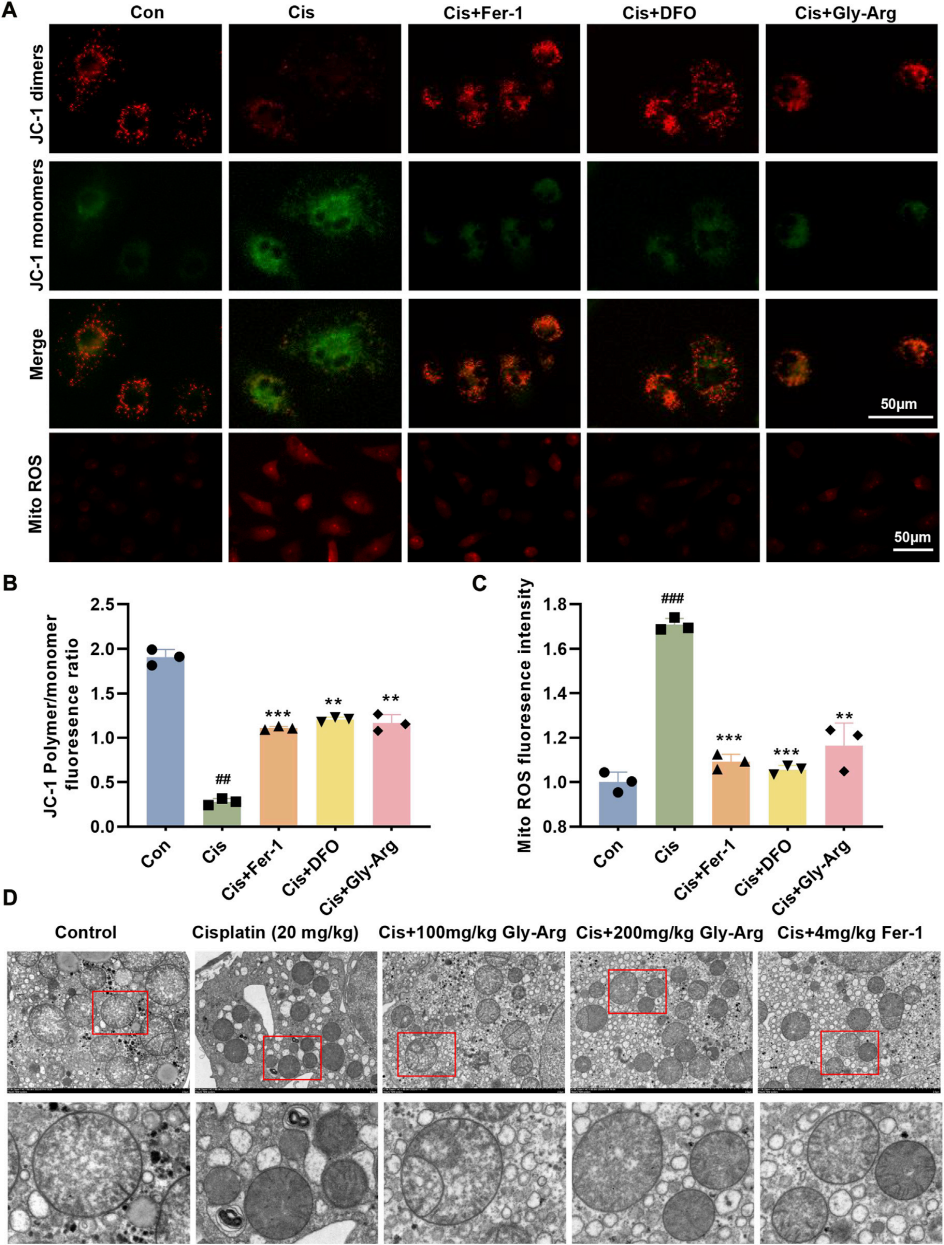
Gly-Arg reverses Cis-induced alterations in the morphology and function of mitochondria. **(A)** A JC-1 probe was used to detect changes in MMP in primary hepatocytes. **(B)** statistical analysis of JC-1 Polymer/monomer fluoresence ratio. **(A)** Fer-1, DFO, and Gly-Arg reduced the level of mito-ROS in primary hepatocytes compared to that of the Cis group. **(C)** statistical analysis of Mito ROS fluoresence intensity. **(D)** Representative TEM images (M: mitochondria; ASS: autolysosomes). ##*p* < 0.01, ###*p* < 0.001 vs. Control; **p < 0.01, ***p < 0.001 vs. Cis.

Mitochondrial damage is also an important marker of ferroptosis (Wu et al., 2021). Therefore, we measured MMP in primary hepatocytes using JC-1 staining. Figure 4A shows that Cis treatment decreased JC-1 dimers and increased JC-1 monomers in primary hepatocytes, indicating that Cis treatment decreased MMP and disrupted mitochondrial function. In contrast, Fer-1, DFO, Gly-Arg and Cis cotreatment reversed these results (4A, 4B). Additionally, Fer-1, DFO, and Gly-Arg reduced the level of mito-ROS in primary hepatocytes compared to that of the Cis-group (4A, 4C). In addition, in the livers of control mice, TEM revealed a clear structure of mitochondrial cristae and few AL (Figure 4D). In the livers of the Cis group, mitochondrial cristae were reduced or disappeared, and the number of ALs was higher than the control group. In contrast, the Gly-Arg group had mildly smaller mitochondria, increased mitochondrial cristae, and less AL than the Cis-group (Figure 4D). Therefore, Gly-Arg resisted Cis-induced ferroptosis by alleviating mitochondrial damage.

### Gly-Arg antagonizes Cis-induced ferroptosis by inhibiting lysosomal-mediated ferritinophagy

**FIGURE 5:**
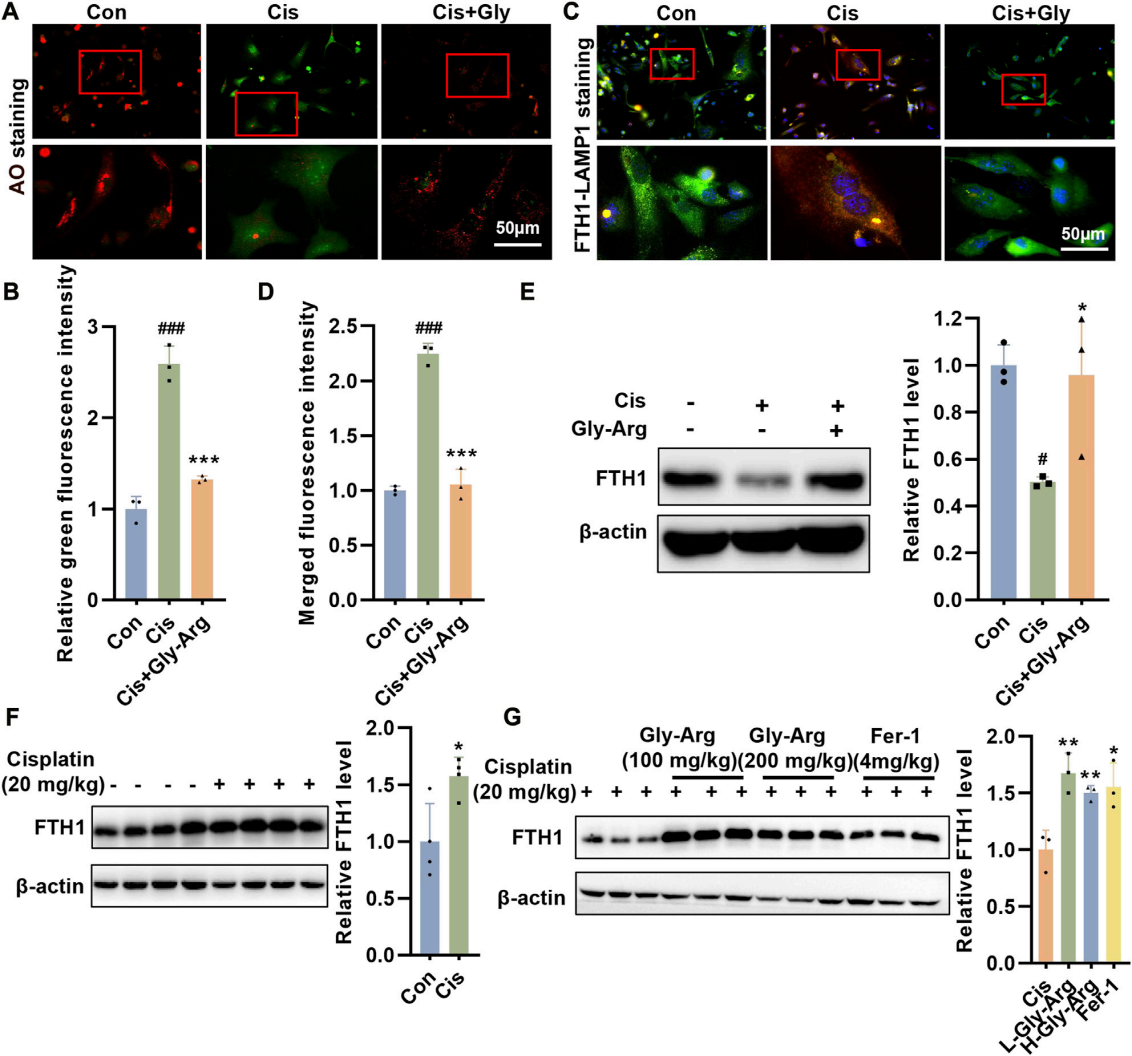
Gly-Arg antagonizes Cis-induced ferroptosis by inhibiting lysosomal-mediated ferritinophagy. **(A, B)** AO staining showed that Gly-Arg pretreatment alleviated LMP, as evidenced by decreased green fluorescence and elevated red fluorescence. **(C, D)** Gly-Arg significantly disrupted the colocalization of LAMP1 and FTH1 in primary hepatocytes compared with Cis treatment alone. **(E)** In primary hepatocytes, the expression of FTH1 decreased after Cis treatment, while Gly-Arg reversed this decrease. **(F)** Compared with that in the control group, the expression of FTH1 was increased in the liver tissues of Cis mice. **(G)** L-Gly-Arg and H-Gly-Arg caused a sustained increase in FTH1 expression compared to the Cis group. *p < 0.05, ***p < 0.001 vs. Control; *p < 0.05, **p < 0.01, ***p < 0.001 vs. Cis.

During ferroptosis, the lysosomal permeability (LMP) of hepatocytes increases (Skouta et al., 2014). When the LMP increases, AO leaks from the lysosome into the cytoplasm, as evidenced by increased green fluorescence and diminished red fluorescence. We noticed that in Cis-treated hepatocytes, green fluorescence increased while red fluorescence decreased, indicating that Cis treatment enhanced LMP (Figures 5A, B). However, after Gly-Arg pretreatment, LMP was alleviated (Figures 5A, B). Free iron is required for lipid peroxidation and ferroptosis, but most of the intracellular iron remains bound to ferritin (Stockwell et al., 2017). During autophagy, ferritin is phagocytosed by lysosomes and then undergoes enzymatic degradation, resulting in the overproduction of free iron (Stockwell et al., 2017). Therefore, we investigated whether lysosomes and ferritin colocalize to elucidate the uptake of ferritin by lysosomes in hepatocytes. IF staining results showed considerable colocalization of FTH1 (green) and lysosome-associated membrane protein 1 (LAMP1) (red) in Cis-treated primary hepatocytes, indicating translocation of ferritin into lysosomes (Figures 5C, D). In contrast, Gly-Arg significantly disrupted the colocalization of the two (Figures 5C, D). In primary hepatocytes, the expression of FTH1 decreased after Cis treatment, while Gly-Arg reversed this decrease (Figure 5E). However, FTH1 expression in liver tissues of mice in the Cis group was opposite to that of primary hepatocytes cultured *in vitro*, which may be related to feedback regulation (Figure 5F). L-Gly-Arg and H-Gly-Arg caused a sustained increase in FTH1 expression compared to the Cis group (Figure 5G). Overall, these results suggest that Gly-Arg antagonizes Cis-induced ferroptosis by inhibiting lysosomal-mediated ferritinophagy.

### Gly-Arg blocks Cis-induced ferroptosis by inhibiting the formation of the BECN1-xCT complex

**FIGURE 6:**
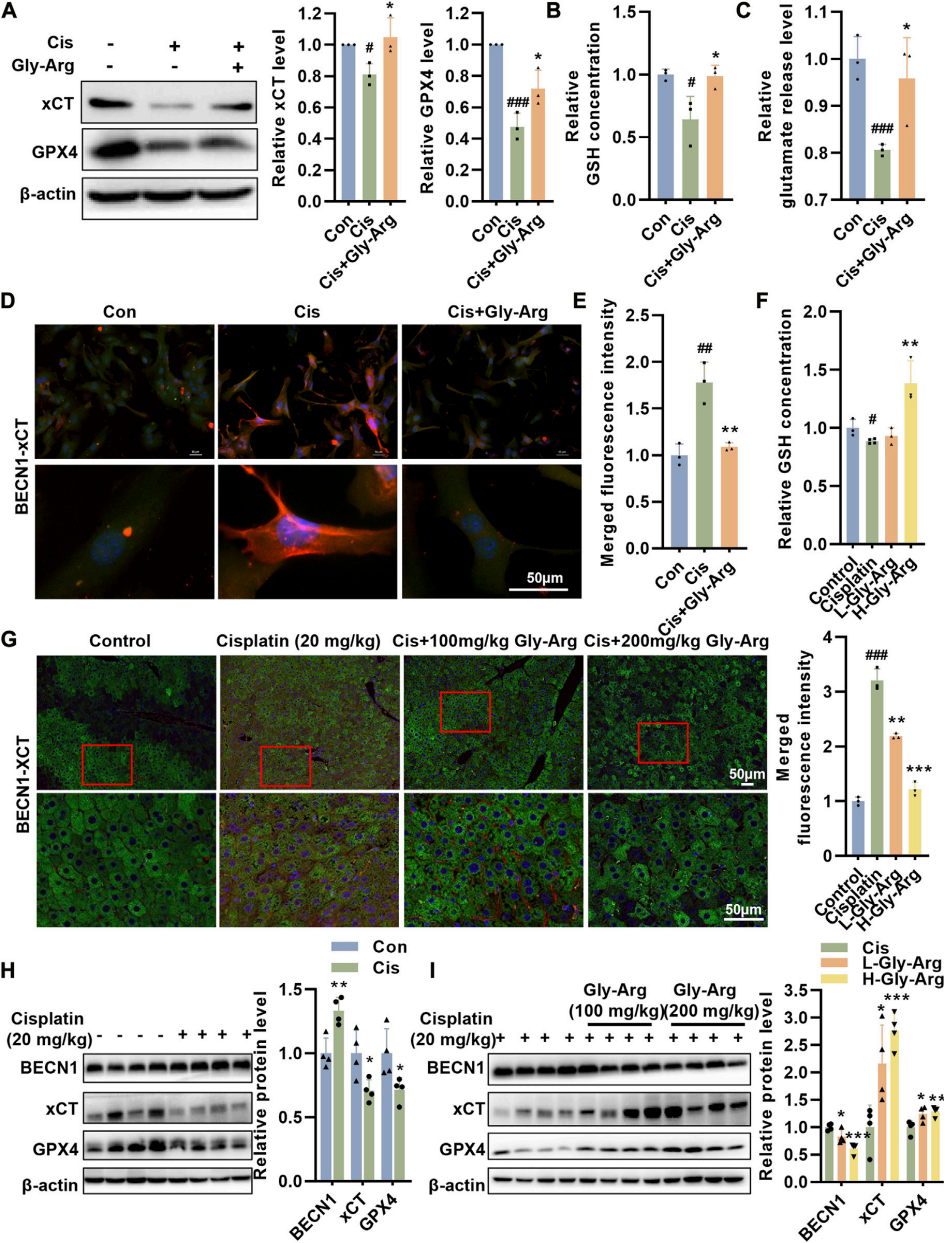
Gly-Arg blocks Cis-induced ferroptosis by inhibiting the formation of the BECN1-xCT complex. **(A)** Western blot assay showed that Gly-Arg reversed Cis-induced reduction of GPX4 and xCT in primary hepatocytes. Gly-Arg alleviated the decreased capacity of GSH **(B)** and glutamate **(C)** release in primary hepatocytes induced by Cis treatment. **(D, E)** Colocalization of xCT-BECN1 was reduced after Gly-Arg treatment compared with that of Cis treatment alone. **(F)** L-Gly-Arg and H-Gly-Arg reversed the Cis-induced decrease in GSH in primary hepatocytes. **(G)** The xCT-BECN1 complex was reduced in both the liver tissues of the L-Gly-Arg and H-Gly-Arg groups compared with those of the Cis group. **(H)** In mouse liver tissues, Cis treatment upregulated BECN1 expression and downregulated xCT and GPX4 expression relative to the control group. **(I)** L-Gly-Arg and H-Gly-Arg were able to reverse this Cis-induced upregulation of BECN1 expression and downregulation of xCT and GPX4 expression in liver tissues. #*p* < 0.05, ##*p* < 0.01, ###*p* < 0.001 vs. Control; *p < 0.05, **p < 0.01, ***p < 0.001 vs. Cis.

The BECN1-xCT complex plays a key role in ferroptosis (Lee et al., 2022; Tan et al., 2022). In the current study, we found that Gly-Arg was able to reverse the Cis-induced increase in BECN1 expression. Therefore, we investigated whether Gly-Arg attenuates ferroptosis by regulating BECN1-mediated changes in the expression and activity of xCT and GPX4. After Cis treatment, the expression of GPX4 and xCT decreased in primary hepatocytes, and Gly-Arg was able to rescue this change (Figure 6A). In addition, we examined GSH and glutamate release in hepatocytes. As we expected, Cis reduced the release of GSH and glutamate in primary hepatocytes, while Gly-Arg was able to alleviate this change (Figures 6B, C). IF staining showed that colocalization between BECN1 and xCT occurred mainly in the cytoplasm of Cis-treated primary hepatocytes (Figures 6D, E), while Gly-Arg pretreatment reduced their colocalization. Similarly, GSH was downregulated in the livers of Cis group mice, whereas L-Gly-Arg and H-Gly-Arg reversed the Cis-induced decrease in GSH (Figure 6F). IF results showed considerable colocalization of xCT (green) and BECN1 (red) in the liver tissue of Cis group mice (Figure 6G). In contrast, xCT-BECN1 colocalization was reduced in both the L-Gly-Arg and H-Gly-Arg groups, and the effect of the high dose was better than that of the low dose (Figure 6G). Meanwhile, in mouse liver tissues, Cis caused upregulation of BECN1 expression and downregulation of xCT and GPX4 expression relative to the control group (Figure 6H). Moreover, L-Gly-Arg and H-Gly-Arg were able to reverse this Cis-induced upregulation of BECN1 expression and downregulation of xCT and GPX4 expression (Figure 6I). Thus, Gly-Arg can block Cis-induced ferroptosis by inhibiting the formation of the BECN1-xCT complex.

### Tat-beclin-1 reverses the protective effect of Gly-Arg against pharmacological Cis-induced liver injury

**FIGURE 7:**
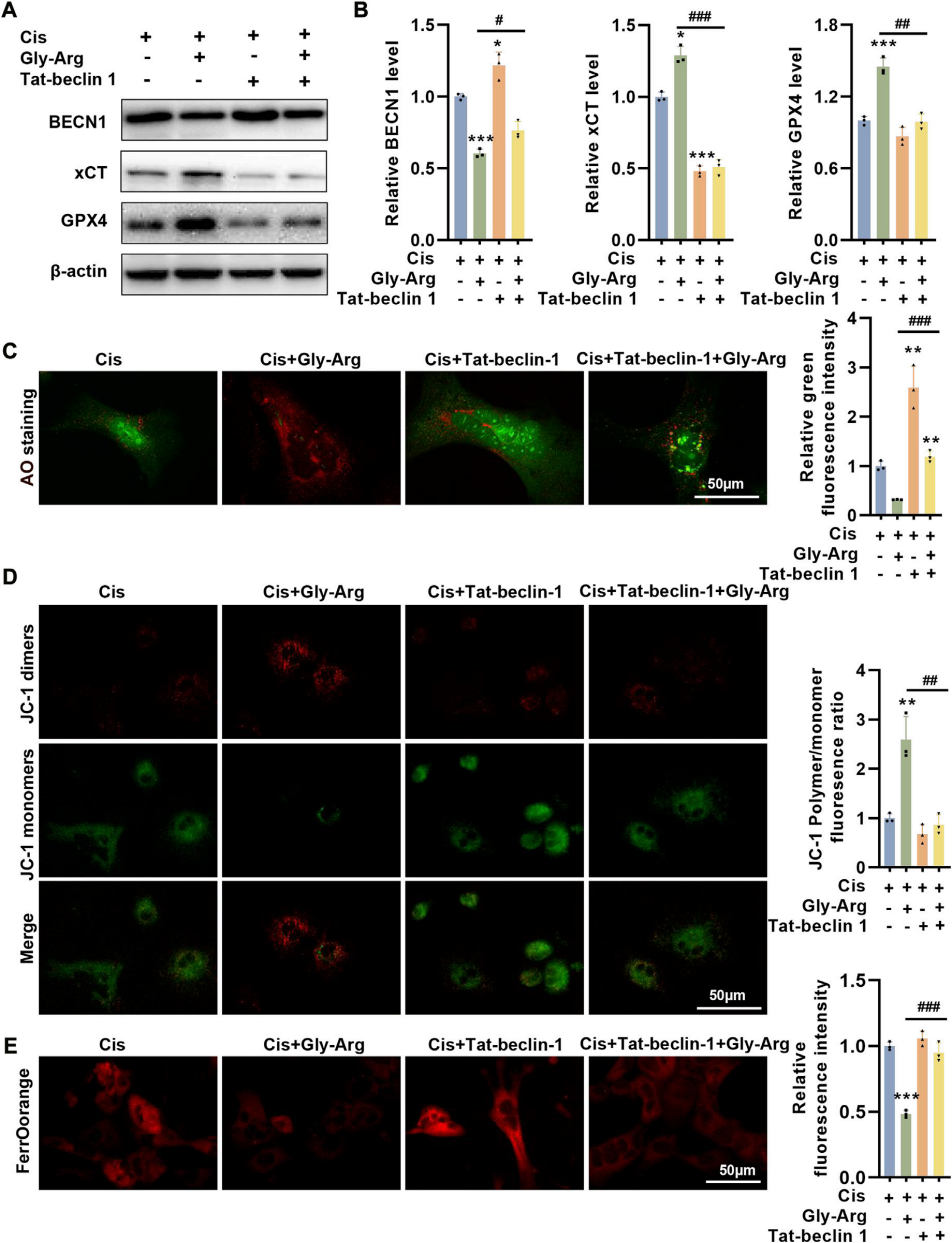
Tat-beclin-1 reverses the protective effect of Gly-Arg against pharmacological Cis-induced liver injury. **(A, B)** In mouse liver primary cells, Cis, Gly-Arg and Tat-beclin 1 cotreatment upregulated BECN1 expression and downregulated xCT and GPX4 expression relative to the Cis and Gly-Arg cotreatment group. **(C)** AO staining showed that Cis, Gly-Arg and Tat-beclin 1 cotreatment showed an increase in LMP relative to the Cis and Gly-Arg cotreatment group. **(D)** JC-1 staining demonstrated that Tat-beclin 1 also reversed the Gly-Arg-induced increase in MMP. **(E)** FerroOrange staining indicated that Tat-beclin 1 also reversed the alleviation of Cis-induced Fe^2+^ accumulation by Gly-Arg. #*p* < 0.05, ##*p* < 0.01, ###*p* < 0.001 vs. Control; *p < 0.05, **p < 0.01, ***p < 0.001 vs. Cis.

We used Tat-beclin 1, an agonist of BECN1, to determine whether it could reverse the protective effect of Gly-Arg. In mouse liver primary cells, Tat-beclin 1 upregulated BECN1 expression and inhibited xCT and GPX4 expression compared to Cis treatment (Figures 7A, B). However, Cis, Gly-Arg and Tat-beclin 1 cotreatment upregulated BECN1 expression and downregulated xCT and GPX4 expression relative to the Cis and Gly-Arg cotreatment group (Figures 7A, B). In addition, Cis, Gly-Arg and Tat-beclin 1 cotreatment showed an increase in LMP relative to the Cis and Gly-Arg cotreatment group (Figure 7C). In addition, Tat-beclin 1 reversed the Gly-Arg-induced increase in MMP (Figure 7D). Moreover, Tat-beclin 1 also reversed the alleviation of Cis-induced Fe^2+^ accumulation by Gly-Arg (Figure 7E). This suggests that BECN1 plays a key role in the protective effect of Gly-Arg against pharmacological Cis-induced liver injury.

## Discussion

Recently, liver injury induced by high-dose cisplatin therapy has received extensive attention (Abd Rashid et al., 2021; Gong et al., 2021). However, the molecular mechanisms responsible for this toxicity are not clear, and therefore, the prevention of cisplatin-induced liver injury has become an important research hotspot in clinical practice (Gong et al., 2021). PCD refers to the autonomous and orderly death process, which is conducive to maintaining the stability of the internal environment, and various pathological liver injuries (such as cirrhosis and liver failure) are closely related to PCD of hepatocytes (Schwabe and Luedde, 2018; Roehlen et al., 2020). Gly is a saponin and its most obvious disadvantage is that Gly forms polymeric gel when exposed to water, which prevents it from being widely used in clinical practice (Zhang et al., 2018). A previous study found that dissolving Gly with arginine in a certain ratio, forming Gly-Arg, could block the formation of Gly polymer gel (Zhang et al., 2018). Moreover, Gly-Arg significantly reduced the degree of cholestatic liver fibrosis in rats, and the mechanism may be related to its inhibition of hepatic stellate cell activation and reduction of oxidative stress and inflammatory response (Zhang et al., 2018). However, whether Gly-Arg ameliorates Cis-induced hepatocyte injury has not yet been reported.

In this paper, we established a mouse model of acute liver injury induced by intraperitoneal injection of Cis. The results showed that the liver tissue of the Cis group showed pathological damage, such as variable morphology and size of hepatocytes, and a large amount of inflammatory cell infiltration. Further investigation revealed that the pathological damage to liver tissue was reduced after Gly-Arg treatment. AST and ALT are important indicators to evaluate the liver function of patients, and when the function of human hepatocytes is affected, a large number of soluble enzymes will gradually enter the blood along with the increase in cell membrane permeability (Shen et al., 2020). LDH also plays an important role in the catabolism and anabolism of amino acids in the liver, and when liver injury occurs, LDH activity increases significantly (Shen et al., 2020). In the present study, AST, ALT and LDH activities were increased in the Cis group, indicating that the Cis-induced liver injury model was successfully established. After treatment with L-Gly-Arg and H-Gly-Arg, AST, ALT and LDH activity were significantly reduced in mice in a dose-dependent manner, indicating that Gly-Arg could inhibit the development of intrahepatic inflammation and improve the abnormal liver function caused by Cis treatment.

At the cellular level, we found that Cis decreased the viability of primary hepatocytes in a concentration-dependent and time-dependent manner, whereas cell viability was significantly increased after Gly-Arg pretreatment. We further explored how Cis mainly induced hepatocyte death. It was found that the apoptosis inhibitor ZVAD, autophagy inhibitor 3-MA, ferroptosis inhibitor Fer-1 and DFO reversed Cis-induced hepatocyte death to some extent. Therefore, we explored whether Gly-Arg ameliorated cisplatin-induced hepatocyte injury by inhibiting apoptosis, autophagy and ferroptosis. Caspases are key regulators of the initiation and execution phases of PCD (Van Opdenbosch and Lamkanfi, 2019). Caspase-3, a cysteine-aspartate protease, is an important effector enzyme in the caspase cascade reaction and is responsible for the enzymatic cleavage of key proteins in the apoptotic pathway (Asadi et al., 2022). In the present study, c-caspase-3 protein expression was elevated after Cis treatment, indicating that apoptosis was present in the livers of the Cis group mice. However, the expression of c-caspase-3 did not change significantly after L-Gly-Arg and H-Gly-Arg treatment. This suggests that Gly-Arg does not ameliorate Cis-induced liver injury by inhibiting apoptosis of hepatocytes.

Next, we explored whether Gly-Arg ameliorates Cis-induced liver injury by inhibiting autophagic cell death. In most cases, autophagy is a stress-induced pathway that promotes cell survival. However, excessive autophagy activation promotes cell death (D'Arcy, 2019). BECN1 plays a key role in the initiation of autophagy and acts synergistically with phosphatidylinositol 3-kinases to control the formation of autophagosomes (Han et al., 2018). LC3 is also an autophagic marker. During autophagy, LC3-I is converted into a membrane-bound form, LC3-II and the LC3-II/LC3-I ratio can reflect the autophagic activity of cells (Tanida et al., 2008). We found that the expression of LC3II/LC3I as well as BECN1 increased significantly after Cis treatment in primary hepatocytes for 24 h. However, the expression of LC3II/LC3I and BECN1 decreased after the addition of Gly-Arg and 3-MA. In addition, LC3II/LC3I was significantly elevated in the livers of mice in the Cis group, while it was reduced in the L-Gly-Arg and H-Gly-Arg groups. These results suggest that Gly-Arg can decrease cell death by blocking the enhanced autophagic activity induced by Cis treatment.

Ferroptosis is an iron-dependent and ROS-mediated form of cell death (Sun et al., 2016). Studies have demonstrated that ferroptosis may be associated with different types of liver diseases, including hepatocellular carcinoma, viral hepatitis C, and nonalcoholic hepatitis (Sun et al., 2016; Tsurusaki et al., 2019; Yamane et al., 2022). Ferroptosis is closely related to intracellular amino acid, iron, and lipid metabolism, with iron overload and lipid peroxidation being the central features (Chen et al., 2021). The liver is an important organ for iron storage in the body, and the accumulation of iron in hepatocytes promotes the development of liver injury by affecting processes such as oxidative stress, the inflammatory response, and fibrosis (Chen et al., 2022). Here, we found that Cis treatment increased oxidative stress as well as inflammatory responses in liver tissues, which caused the development of liver injury. Furthermore, the relative fluorescence intensities of the peroxidation products 4-HNE, Fe^2+^, and lipid ROS were higher in the Cis group than in the control group, while Gly-Arg reduced the accumulation of the peroxidation products in liver tissues.

Ferroptosis is associated with severe damage to mitochondrial morphology and energy metabolism (Battaglia et al., 2020). MMP produced by the mitochondrial proton pump is essential for ATP production by coupling with oxidative phosphorylation (Zorova et al., 2018). Different ferroptosis inducers, including cystine starvation and amino acid-free medium, can induce mitochondrial hyperpolarization and destroy MMP (Gao et al., 2019). Additionally, mito-ROS promotes ferroptosis (Wu et al., 2021). For example, in nerve cells, RSL3-induced ferroptosis can be blocked by the mito-ROS scanner MitoQ (mitoquinone) (Jelinek et al., 2018). In this study, we found that Cis treatment significantly decreased MMP and increased mito-ROS production in primary hepatocytes. To explore whether Gly-Arg improves ferroptosis by decreasing mito-ROS, DFO (an Fe^2+^ chelator) and Fer-1 (a lipid peroxidation scavenger) were selected. DFO and Fer-1 reduced the Cis-induced reduction of MMP and elevation of mito-ROS compared to Cis treatment alone. In contrast, Gly-Arg reduced Cis-induced changes as well. Another major feature of ferroptosis includes a reduction in mitochondrial fragmentation, a decrease or loss of mitochondrial cristae, and rupture of the outer membrane (Wu, H. et al., 2021). TEM revealed that the liver mitochondrial cristae were reduced or disappeared and that the outer mitochondrial membrane ruptured in Cis-treated mice, while the application of Gly-Arg effectively prevented the ultrastructural alteration of liver cell mitochondria. These results indicate that Gly-Arg can improve Cis-induced hepatocyte injury by reducing mito-ROS and mitochondrial function.

Autophagy is an important mechanism for maintaining cellular homeostasis that is dependent on lysosomes (Levine and Kroemer, 2019). Lysosomes are single-membrane organelles containing acidic hydrolases that not only play an important role in degradation and recycling but are also responsible for maintaining iron homeostasis (Rizzollo et al., 2021). Lysosomes are the first organelles to receive extracellular iron via the endocytic pathway and are responsible for its reduction to Fe^2+^. These high concentrations of reactive iron make lysosomes more sensitive to oxidative stress (Rizzollo et al., 2021). Hence, slight oxidative stress in the cell can trigger lysosomal membrane instability. Lysosomal membrane instability can cause the release of lysosomal hydrolases into the cytoplasm, and lysosome membrane permeabilization (LMP) occurs, leading to abnormal mitochondrial function as well as cellular damage (Rizzollo et al., 2021). Our results found that Cis treatment caused an increase in LMP in primary hepatocytes, indicating that lysosomes were exposed to Cis-induced oxidative stress. Under normal physiological conditions, lysosomes consume iron by forming chelates, reducing the sensitivity of lysosomes to oxidative stress (Pegg, 2016; van Veen et al., 2020). However, activation of autophagy, particularly ferritinophagy, can degrade ferritin to increase intracellular iron content and subsequently lead to oxidative damage in response to Fenton (Qin et al., 2021). This process is mediated by nuclear receptor coactivator 4 (NCOA4), which selectively binds FTH1 in autophagosomes and delivers it to lysosomes, leading to Fe^2+^ release (Santana-Codina et al., 2021). In primary hepatocytes, the colocalization of FTH1 with the lysosomal marker protein LAMP1 was significantly increased after Cis treatment, indicating that FTH1 is degraded through a lysosome-dependent pathway. Moreover, at the protein level, we found that Cis treatment reduced the expression of FTH1 in primary hepatocytes. After Gly-Arg treatment, the colocalization of FTH1 with LAMP1 was reduced, and the protein expression of FTH1 was increased, suggesting that Gly-Arg promotes the storage of excess Fe^2+^ in ferritin, thereby alleviating ferroptosis. However, in the livers of the Cis group, the expression of FTH1 was elevated compared with that of the control. This may be due to the regulation of a complex feedback mechanism. Guo et al. found that in human fibrosarcoma cell lines, the transcription and expression of endogenous FTH1 were elevated by an increase in intracellular free iron levels when ferroptosis occurred (Gao et al., 2016). Consistent with the *in vitro* results, the expression of FTH1 in mouse liver tissues was also elevated after L-Gly-Arg and H-Gly-Arg treatment compared with those of the Cis group. Overall, Gly-Arg blocks Cis-induced ferritinophagy to some extent by inhibiting the autophagic degradation of FTH1.

BECN1 is another central effector molecule of autophagy (Kang et al., 2018). A recent study found that BECN1-xCT complex formation is essential for ferroptosis (Kang et al., 2018; Song et al., 2018). Song et al. reported that AMPK-mediated phosphorylation of the BECN1 Ser90/93/96 sites is required for BECN1-xCT complex formation and lipid peroxidation (Song et al., 2018). More importantly, BECN1 expression only affects xCT inhibitor-induced ferroptosis and does not affect intracellular iron accumulation or the expression of genes related to iron metabolism (Song et al., 2018). In addition, knockdown of BECN1 was found to improve early brain injury (EBI) after subarachnoid hemorrhage (SAH) by increasing xCT activity and inhibiting lipid peroxidation (Guo et al., 2019). In head and neck cancer cells, knockdown of the cytosolic iron chaperone poly (rC)-binding protein 1 (PCBP1) increased BECN1 mRNA stability and inhibited xCT expression and viability, thereby promoting ferroptosis (Lee et al., 2022). These results suggest that BECN1 is a common target of ferroptosis and autophagy regulation. Therefore, we explored whether the BECN1-xCT complex regulates Cis-induced ferroptosis in primary hepatocytes. The experimental results showed that BECN1 expression was elevated, but xCT activity and expression were decreased in the Cis group compared with those of the control group. The IF results showed that the colocalization of BECN1 and xCT was significantly enhanced in primary hepatocytes of the Cis group. These results suggest that Cis treatment may inhibit xCT activity by promoting the formation of a complex between BECN1 and xCT, leading to a decrease in intracellular GSH content inhibition of GPX4 expression and ultimately triggering ferroptosis. In contrast, Gly-Arg treatment decreased the colocalization of BECN1 and xCT, which then decreased the expression of xCT/GPX4. To further determine the molecular mechanism by which Gly-Arg ameliorates Cis-induced autophagy and ferroptosis, we activated BECN1 expression with Tat-beclin 1 in primary hepatocytes. Our results showed that increased BECN1 expression decreased xCT and GPX4 protein levels, increased lysosomal permeability, decreased MMP, and elevated Fe^2+^ levels. More importantly, the effect of Gly-Arg against ferroptosis could be reversed to some extent by the increased expression of BECN1. Accordingly, we speculate that Gly-Arg may drive xCT/GPX4-mediated anti-ferroptosis effects by reducing BECN1 expression to ameliorate Cis-induced liver injury.

**FIGURE 8:**
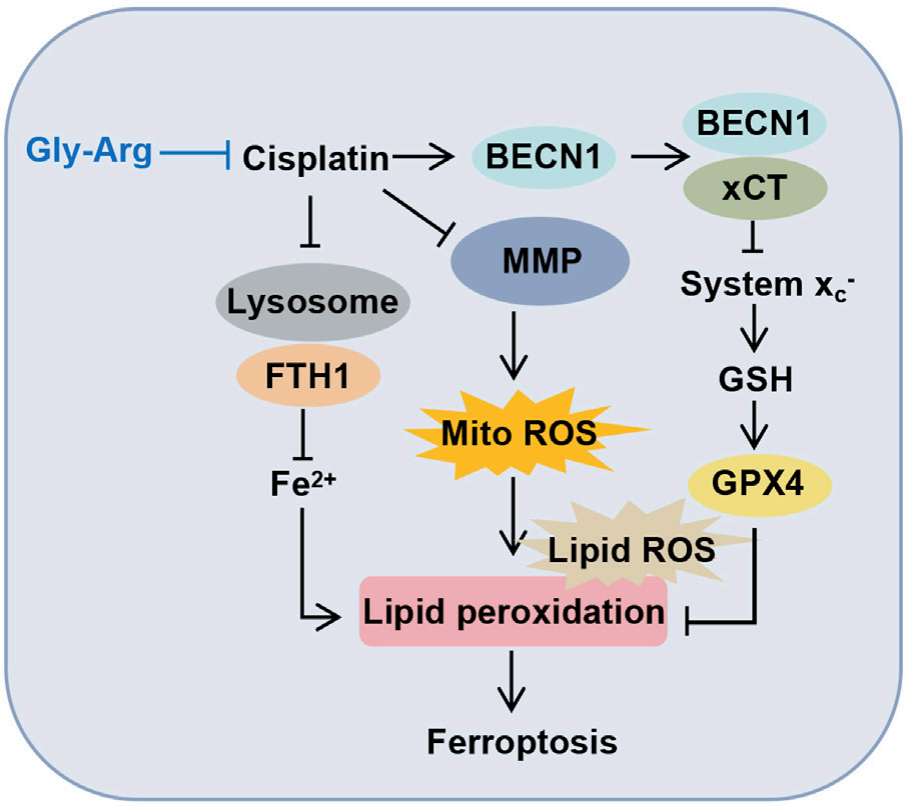
Molecular mechanism diagram of Gly-Arg improving Cis-induced liver injury.

In summary, our data confirm for the first time that Gly-Arg plays a protective role against Cis-induced liver injury by inhibiting the formation of the BECN1-xCT complex and antagonizing Cis-induced autophagy and ferroptosis in hepatocytes (Figure 8). This study provides strong evidence for the success of Gly-Arg as an effective and safe agent for neutralizing the disadvantages of Cis treatment.

## Figures and Tables

**Table:** 

**3-MA**	3-Methyladenine
**AL**	autolysosomes
**4-ALT**	glutamate aminotransferase
**ALT/GPT**	alanine aminotransferase
**AMPK**	AMP-activated catalytic subunit alpha 1
**4-AST**	glutamate aminotransferase
**AST/GOT**	aspartate aminotransferase
**AO**	Acridine orange
**BECN1**	Beclin1
**CAT**	Catalase
**Cis**	Cisplatin
**CQ**	chloroquine
**DFO**	deferiprone
**DMSO**	Dimethyl sulfoxide
**EBI**	early brain injury
**FBS**	fetal bovine serum
**Gly**	Glycyrrhizin
**Gly-Arg**	glycyrrhizin arginine salt
**GPX4**	glutathione peroxidase-4
**GSH**	Glutathione
**GSH-PX**	glutathione peroxidase
**H&E**	Hematoxylin and eosin staining
**JC-1**	mitochondrial membrane potential
**LAMP1**	ferritin heavy chain 1
**LDH**	lactate dehydrogenase
**LMP**	lysosomal permeability
**MDA**	malondialdehyde
**Mito-ROS**	mitochondrial reactive oxygen species
**MMP**	mitochondrial membrane potential
**MTT**	3-(4,5-Dimethylthiazol-2-yl)-2,5-diphenyltetrazolium bromide
**Nec-1**	necrostatin-1
**PCD**	Programmed cell death
**PCBP1**	poly (rC)-binding protein 1
**ROS**	reactive oxygen species
**SAH**	subarachnoid hemorrhage
**SLC7A11, xCT**	solute carrier family 7 member 11
**SPF**	specific pathogen-free
**TEM**	Transmission electron microscopy
**ZVAD**	Z-VAD-FMK

## Data Availability

The original contributions presented in the study are included in the article/Supplementary Material, further inquiries can be directed to the corresponding authors.

## References

[B1] Abd Rashid N. Abd Halim S. A. S. Teoh S. L. Budin S. B. Hussan F. Adib Ridzuan N. R. (2021). The role of natural antioxidants in cisplatin-induced hepatotoxicity. Biomed. Pharmacother. 144, 112328. 10.1016/j.biopha.2021.112328 34653753

[B2] Asadi M. Taghizadeh S. Kaviani E. Vakili O. Taheri-Anganeh M. Tahamtan M. (2022). Caspase-3: structure, function, and biotechnological aspects. Biotechnol. Appl. Biochem. 69, 1633–1645. 10.1002/bab.2233 34342377

[B3] Battaglia A. M. Chirillo R. Aversa I. Sacco A. Costanzo F. Biamonte F. (2020). Ferroptosis and cancer: mitochondria meet the "iron maiden" cell death. Cells 9, 1505. 10.3390/cells9061505 32575749 PMC7349567

[B4] Cai C. Guo Z. Chang X. Li Z. Wu F. He J. (2022). Empagliflozin attenuates cardiac microvascular ischemia/reperfusion through activating the AMPKα1/ULK1/FUNDC1/mitophagy pathway. Redox Biol. 52, 102288. 10.1016/j.redox.2022.102288 35325804 PMC8938627

[B5] Chen J. Li X. Ge C. Min J. Wang F. (2022). The multifaceted role of ferroptosis in liver disease. Cell. death Differ. 29, 467–480. 10.1038/s41418-022-00941-0 35075250 PMC8901678

[B6] Chen X. Li J. Kang R. Klionsky D. J. Tang D. (2021). Ferroptosis: machinery and regulation. Autophagy 17, 2054–2081. 10.1080/15548627.2020.1810918 32804006 PMC8496712

[B7] D'Arcy M. S. (2019). Cell death: A review of the major forms of apoptosis, necrosis and autophagy. Cell. Biol. Int. 43, 582–592. 10.1002/cbin.11137 30958602

[B8] El Nashar E. M. Alghamdi M. A. Alasmari W. A. Hussein M. M. A. Hamza E. Taha R. I. (2021). Autophagy promotes the survival of adipose mesenchymal stem/stromal cells and enhances their therapeutic effects in cisplatin-induced liver injury via modulating TGF-β1/smad and PI3K/AKT signaling pathways. Cells 10, 2475. 10.3390/cells10092475 34572126 PMC8470434

[B9] Fang Y. Chen X. Tan Q. Zhou H. Xu J. Gu Q. (2021). Inhibiting ferroptosis through disrupting the NCOA4-FTH1 interaction: A new mechanism of action. ACS central Sci. 7, 980–989. 10.1021/acscentsci.0c01592 PMC8227600 34235259

[B10] Fukuoka K. Inagaki A. Nakamura Y. Matsumura M. Yoshida S. Imura T. (2017). The optimization of short-term hepatocyte preservation before transplantation. Transpl. Direct 3, e176. 10.1097/TXD.0000000000000687 PMC5498017 28706979

[B11] Gao M. Monian P. Pan Q. Zhang W. Xiang J. Jiang X. (2016). Ferroptosis is an autophagic cell death process. Cell. Res. 26, 1021–1032. 10.1038/cr.2016.95 27514700 PMC5034113

[B12] Gao M. Yi J. Zhu J. Minikes A. M. Monian P. Thompson C. B. (2019). Role of mitochondria in ferroptosis. Mol. Cell. 73, 354–363. 10.1016/j.molcel.2018.10.042 30581146 PMC6338496

[B13] Gong S. Feng Y. Zeng Y. Zhang H. Pan M. He F. (2021). Gut microbiota accelerates cisplatin-induced acute liver injury associated with robust inflammation and oxidative stress in mice. J. Transl. Med. 19, 147. 10.1186/s12967-021-02814-5 33849559 PMC8045234

[B14] Griffioen A. W. Nowak-Sliwinska P. (2022). Programmed cell death lives. Apoptosis Int. J. Program. Cell. death 27, 619–621. 10.1007/s10495-022-01758-5 PMC9361233 35943678

[B15] Guo Y. Liu X. Liu D. Li K. Wang C. Liu Y. (2019). Inhibition of BECN1 suppresses lipid peroxidation by increasing system Xc(-) activity in early brain injury after subarachnoid hemorrhage. J. Mol. Neurosci. 67, 622–631. 10.1007/s12031-019-01272-5 30719640

[B16] Han T. Guo M. Gan M. Yu B. Tian X. Wang J. B. (2018). TRIM59 regulates autophagy through modulating both the transcription and the ubiquitination of BECN1. Autophagy 14, 2035–2048. 10.1080/15548627.2018.1491493 30231667 PMC6984771

[B17] Hou W. Xie Y. Song X. Sun X. Lotze M. T. Zeh H. J. (2016). Autophagy promotes ferroptosis by degradation of ferritin. Autophagy 12, 1425–1428. 10.1080/15548627.2016.1187366 27245739 PMC4968231

[B18] Jelinek A. Heyder L. Daude M. Plessner M. Krippner S. Grosse R. (2018). Mitochondrial rescue prevents glutathione peroxidase-dependent ferroptosis. Free Radic. Biol. Med. 117, 45–57. 10.1016/j.freeradbiomed.2018.01.019 29378335

[B19] Jiang X. Stockwell B. R. Conrad M. (2021). Ferroptosis: mechanisms, biology and role in disease. Nat. Rev. Mol. Cell. Biol. 22, 266–282. 10.1038/s41580-020-00324-8 33495651 PMC8142022

[B20] Kang R. Zhu S. Zeh H. J. Klionsky D. J. Tang D. (2018). BECN1 is a new driver of ferroptosis. Autophagy 14, 2173–2175. 10.1080/15548627.2018.1513758 30145930 PMC6984768

[B21] Kurt N. Turkeri O. N. Suleyman B. Bakan N. (2021). The effect of taxifolin on high-dose-cisplatin-induced oxidative liver injury in rats. Adv. Clin. Exp. Med. official organ Wroclaw Med. Univ. 30, 1025–1030. 10.17219/acem/138318 34435476

[B22] Lee J. You J. H. Roh J. L. (2022). Poly(rC)-binding protein 1 represses ferritinophagy-mediated ferroptosis in head and neck cancer. Redox Biol. 51, 102276. 10.1016/j.redox.2022.102276 35290903 PMC8921323

[B23] Levine B. Kroemer G. (2019). Biological functions of autophagy genes: A disease perspective. Cell. 176, 11–42. 10.1016/j.cell.2018.09.048 30633901 PMC6347410

[B24] Li J. Shi J. Sun Y. Zheng F. (2018). Glycyrrhizin, a potential drug for autoimmune encephalomyelitis by inhibiting high-mobility group box 1. DNA Cell. Biol. 37, 941–946. 10.1089/dna.2018.4444 30325653

[B25] Liu J. Kuang F. Kroemer G. Klionsky D. J. Kang R. Tang D. (2020). Autophagy-dependent ferroptosis: machinery and regulation. Cell. Chem. Biol. 27, 420–435. 10.1016/j.chembiol.2020.02.005 32160513 PMC7166192

[B26] Matsumura M. Imura T. Inagaki A. Ogasawara H. Fukuoka K. Fathi I. (2019). A simple and useful predictive assay for evaluating the quality of isolated hepatocytes for hepatocyte transplantation. Sci. Rep. 9, 6166. 10.1038/s41598-019-42720-x 30992529 PMC6467914

[B27] Mizushima N. Yoshimori T. Levine B. (2010). Methods in mammalian autophagy research. Cell. 140, 313–326. 10.1016/j.cell.2010.01.028 20144757 PMC2852113

[B28] Mou Y. Wang J. Wu J. He D. Zhang C. Duan C. (2019). Ferroptosis, a new form of cell death: opportunities and challenges in cancer. J. Hematol. Oncol. 12, 34. 10.1186/s13045-019-0720-y 30925886 PMC6441206

[B29] Niu C. Ma M. Han X. Wang Z. Li H. (2017). Hyperin protects against cisplatin-induced liver injury in mice. Acta cir. bras. 32, 633–640. 10.1590/s0102-865020170080000005 28902939

[B30] Pegg A. E. (2016). Functions of polyamines in mammals. J. Biol. Chem. 291, 14904–14912. 10.1074/jbc.R116.731661 27268251 PMC4946908

[B31] Qin X. Zhang J. Wang B. Xu G. Yang X. Zou Z. (2021). Ferritinophagy is involved in the zinc oxide nanoparticles-induced ferroptosis of vascular endothelial cells. Autophagy 17, 4266–4285. 10.1080/15548627.2021.1911016 33843441 PMC8726675

[B32] Rizzollo F. More S. Vangheluwe P. Agostinis P. (2021). The lysosome as a master regulator of iron metabolism. Trends Biochem. Sci. 46, 960–975. 10.1016/j.tibs.2021.07.003 34384657

[B33] Roehlen N. Crouchet E. Baumert T. F. (2020). Liver fibrosis: mechanistic concepts and therapeutic perspectives. Cells 9, 875. 10.3390/cells9040875 32260126 PMC7226751

[B34] Santana-Codina N. Gikandi A. Mancias J. D. (2021). The role of NCOA4-mediated ferritinophagy in ferroptosis. Adv. Exp. Med. Biol. 1301, 41–57. 10.1007/978-3-030-62026-4_4 34370287

[B35] Schwabe R. F. Luedde T. (2018). Apoptosis and necroptosis in the liver: A matter of life and death. Nat. Rev. Gastroenterology hepatology 15, 738–752. 10.1038/s41575-018-0065-y 30250076 PMC6490680

[B36] Shen Y. Shen X. Cheng Y. Liu Y. (2020). Myricitrin pretreatment ameliorates mouse liver ischemia reperfusion injury. Int. Immunopharmacol. 89, 107005. 10.1016/j.intimp.2020.107005 33045574

[B37] Skouta R. Dixon S. J. Wang J. Dunn D. E. Orman M. Shimada K. (2014). Ferrostatins inhibit oxidative lipid damage and cell death in diverse disease models. J. Am. Chem. Soc. 136, 4551–4556. 10.1021/ja411006a 24592866 PMC3985476

[B38] Song X. Zhu S. Chen P. Hou W. Wen Q. Liu J. (2018). AMPK-mediated BECN1 phosphorylation promotes ferroptosis by directly blocking system Xc(-) activity. Curr. Biol. 28, 2388–2399. 10.1016/j.cub.2018.05.094 30057310 PMC6081251

[B39] Stockwell B. R. Friedmann Angeli J. P. Bayir H. Bush A. I. Conrad M. Dixon S. J. (2017). Ferroptosis: A regulated cell death nexus linking metabolism, redox biology, and disease. Cell. 171, 273–285. 10.1016/j.cell.2017.09.021 28985560 PMC5685180

[B40] Storey S. M. McIntosh A. L. Huang H. Martin G. G. Landrock K. K. Landrock D. (2012). Loss of intracellular lipid binding proteins differentially impacts saturated fatty acid uptake and nuclear targeting in mouse hepatocytes. Am. J. Physiol. Gastrointest. Liver Physiol. 303, G837–G850. 10.1152/ajpgi.00489.2011 22859366 PMC3469595

[B41] Sun X. Ou Z. Chen R. Niu X. Chen D. Kang R. (2016). Activation of the p62-Keap1-NRF2 pathway protects against ferroptosis in hepatocellular carcinoma cells. Hepatology 63, 173–184. 10.1002/hep.28251 26403645 PMC4688087

[B42] Tan Y. Huang Y. Mei R. Mao F. Yang D. Liu J. (2022). HucMSC-derived exosomes delivered BECN1 induces ferroptosis of hepatic stellate cells via regulating the xCT/GPX4 axis. Cell. death Dis. 13, 319. 10.1038/s41419-022-04764-2 35395830 PMC8993870

[B43] Tanida I. Ueno T. Kominami E. (2008). LC3 and autophagy. Methods Mol. Biol. 445, 77–88. 10.1007/978-1-59745-157-4_4 18425443

[B44] Tsurusaki S. Tsuchiya Y. Koumura T. Nakasone M. Sakamoto T. Matsuoka M. (2019). Hepatic ferroptosis plays an important role as the trigger for initiating inflammation in nonalcoholic steatohepatitis. Cell. death Dis. 10, 449. 10.1038/s41419-019-1678-y 31209199 PMC6579767

[B45] Van Opdenbosch N. Lamkanfi M. (2019). Caspases in cell death, inflammation, and disease. Immunity 50, 1352–1364. 10.1016/j.immuni.2019.05.020 31216460 PMC6611727

[B46] van Veen S. Martin S. Van den Haute C. Benoy V. Lyons J. Vanhoutte R. (2020). ATP13A2 deficiency disrupts lysosomal polyamine export. Nature 578, 419–424. 10.1038/s41586-020-1968-7 31996848

[B47] Wu H. Wang F. Ta N. Zhang T. Gao W. (2021). The multifaceted regulation of mitochondria in ferroptosis. Life 11, 222. 10.3390/life11030222 33801920 PMC8001967

[B48] Xie Y. Hou W. Song X. Yu Y. Huang J. Sun X. (2016). Ferroptosis: process and function. Cell. death Differ. 23, 369–379. 10.1038/cdd.2015.158 26794443 PMC5072448

[B49] Yamane D. Hayashi Y. Matsumoto M. Nakanishi H. Imagawa H. Kohara M. (2022). FADS2-dependent fatty acid desaturation dictates cellular sensitivity to ferroptosis and permissiveness for hepatitis C virus replication. Cell. Chem. Biol. 29, 799–810.e4. 10.1016/j.chembiol.2021.07.022 34520742 PMC8913804

[B50] Zhang H. Lin Y. Zhen Y. Hu G. Meng X. Li X. (2018). Therapeutic effect of glycyrrhizin arginine salt on rat cholestatic cirrhosis and its mechanism. Am. J. Chin. Med. 46, 1111–1127. 10.1142/S0192415X18500581 29976082

[B51] Zhang Z. Guo M. Li Y. Shen M. Kong D. Shao J. (2020). RNA-binding protein ZFP36/TTP protects against ferroptosis by regulating autophagy signaling pathway in hepatic stellate cells. Autophagy 16, 1482–1505. 10.1080/15548627.2019.1687985 31679460 PMC7469536

[B52] Zorova L. D. Popkov V. A. Plotnikov E. Y. Silachev D. N. Pevzner I. B. Jankauskas S. S. (2018). Mitochondrial membrane potential. Anal. Biochem. 552, 50–59. 10.1016/j.ab.2017.07.009 28711444 PMC5792320

