# Supplementary figures and images for: Glycyrrhizin arginine salt protects against cisplation-induced acute liver injury by repressing BECN1-mediated ferroptosis
Source: Front Pharmacol. 2023 Sep 6;14:1219486. doi: 10.3389/fphar.2023.1219486 (PMC10511756; doi:10.3389/fphar.2023.1219486)

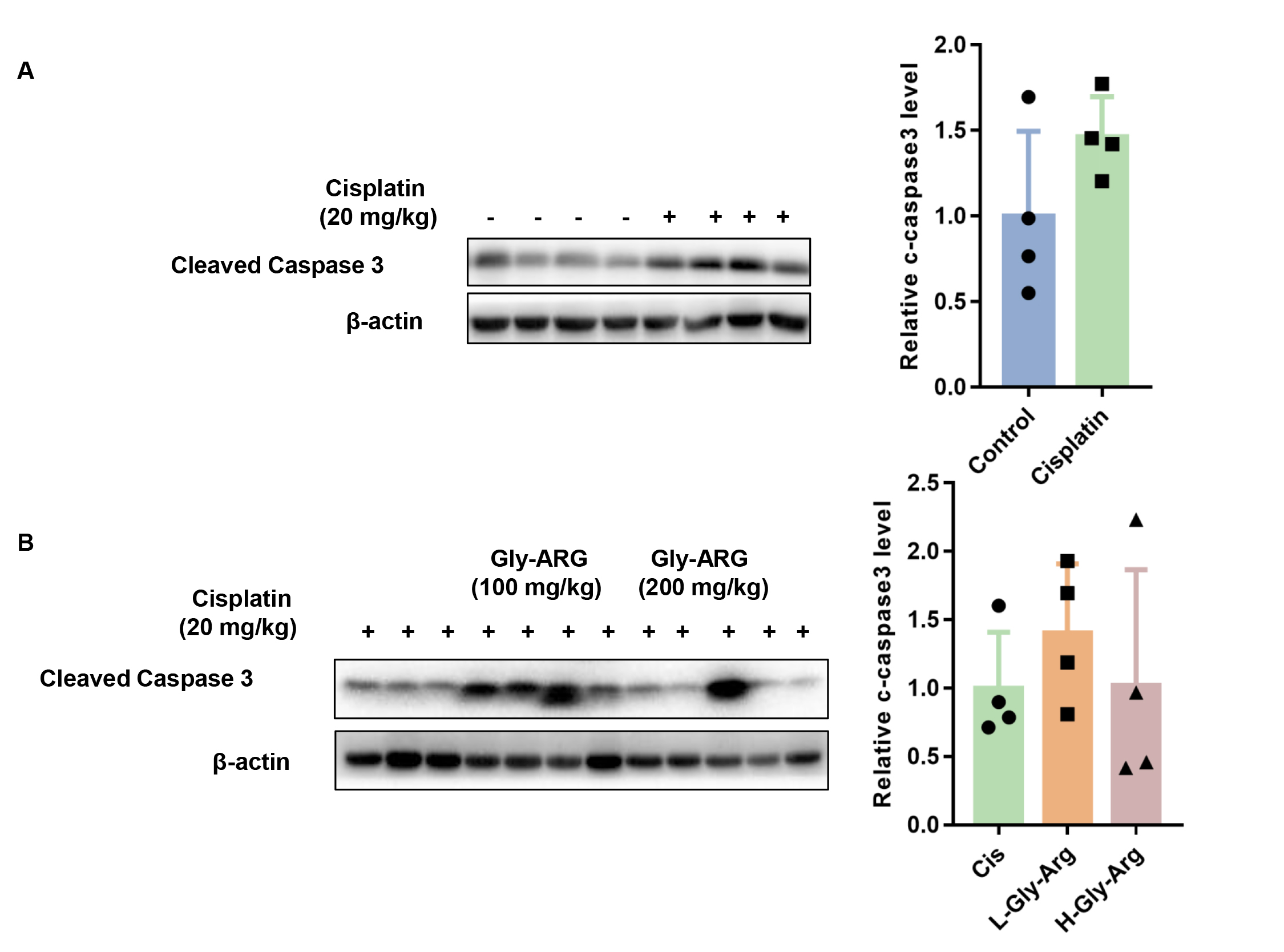

Supplement: Supplementary file 1 [file Image2.jpg]

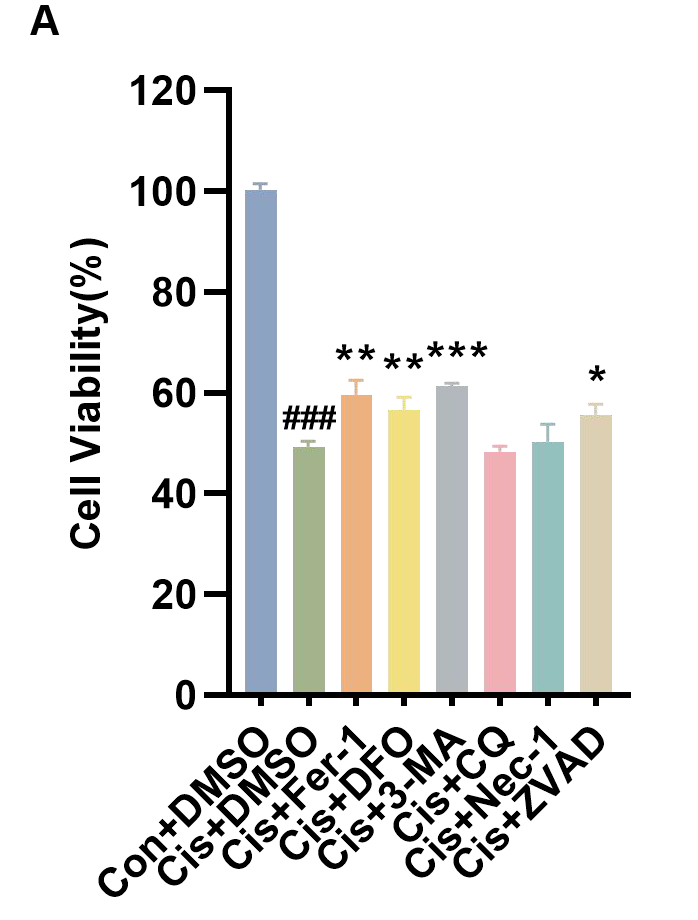

Supplement: Supplementary file 2 [file Image1.tif]
